# Does Extreme Language Control Training Improve Cognitive Control? A Comparison of Professional Interpreters, L2 Teachers and Monolinguals

**DOI:** 10.3389/fpsyg.2018.01998

**Published:** 2018-10-23

**Authors:** Lize Van der Linden, Eowyn Van de Putte, Evy Woumans, Wouter Duyck, Arnaud Szmalec

**Affiliations:** ^1^Psychological Sciences Research Institute, Université catholique de Louvain, Louvain-la-Neuve, Belgium; ^2^Department of Experimental Psychology, Ghent University, Ghent, Belgium; ^3^Institute of Neuroscience, Université catholique de Louvain, Louvain-la-Neuve, Belgium

**Keywords:** bilingualism, interpreting, cognitive control, language control, bilingual experience

## Abstract

There is currently a lively debate in the literature whether bilingualism leads to enhanced cognitive control or not. Recent evidence suggests that knowledge of more than one language does not always suffice for the manifestation of a bilingual cognitive control advantage. As a result, ongoing research has focused on modalities of bilingual language use that may interact with the bilingual advantage. In this study, we explored the cognitive control performance of simultaneous interpreters. These highly proficient bilinguals comprehend information in one language while producing in the other language, which is a complex skill requiring high levels of language control. In a first experiment, we compared professional interpreters to monolinguals. Data were collected on interference suppression (flanker task), prepotent response inhibition (Simon task), and short-term memory (digit span task). The results showed that the professional interpreters performed similarly to the monolinguals on all measures. In Experiment 2, we compared professional interpreters to monolinguals and second language teachers. Data were collected on interference suppression (advanced flanker task), prepotent response inhibition (advanced flanker task), attention (advanced flanker task), short-term memory (Hebb repetition paradigm), and updating (*n*-back task). We found converging evidence for our finding that experience in interpreting may not lead to superior interference suppression, prepotent response inhibition, and short-term memory. In fact, our results showed that the professional interpreters performed similarly to both the monolinguals and the second language teachers on all tested cognitive control measures. We did, however, find anecdotal evidence for a (small) advantage in short-term memory for interpreters relative to monolinguals when analyzing composite scores of both experiments together. Taken together, the results of the current study suggest that interpreter experience does not necessarily lead to general cognitive control advantages. However, there may be small interpreter advantages in short-term memory, suggesting that this might be an important cognitive control aspect of simultaneous interpreting. The results are discussed in the light of ongoing debates about bilingual cognitive control advantages.

## Introduction

Recent research has shown that certain cognitively demanding activities, such as playing video games, playing music, and mastering chess, may be beneficial to human cognition, beyond the domain of practice (e.g., Reingold et al., 2001; Bialystok, 2006; Schroeder et al., 2016). Gaining expertise in a certain skill may lead to a transfer of the acquired abilities to other behaviors that involve the same processes, often related to cognitive control. Cognitive control is an umbrella term for the cognitive processes that guide goal-directed behavior. Knowing and using a second language (L2) in daily life, or bilingualism (Grosjean, 2010), may also have beneficial effects on cognition. Bilinguals outperform monolinguals in learning novel words (e.g., Kaushanskaya and Marian, 2009; Nair et al., 2017). Similarly, bilinguals outperform monolinguals on non-verbal tasks that require different cognitive control processes, like conflict resolution, attention, shifting, updating, and working memory, for example (e.g., Bialystok et al., 2006, 2008; Costa et al., 2008; Prior and Macwhinney, 2010; Luo et al., 2013). One explanation for these bilingual advantages is that using multiple languages requires a mechanism to select (words in) the target language while avoiding interference from the other known language. There is in fact compelling evidence that both languages of bilinguals are always simultaneously active in their mind (e.g., Colomé, 2001; Duyck and Warlop, 2009; Van Assche et al., 2009). Bilinguals therefore need to control (inhibit) activation of the non-target language to use the intended language (Green, 1998). The mechanisms that allow this language control are believed to be domain-general and hence, not specific to the linguistic domain. Using multiple languages in daily life might therefore train domain-general cognitive control, in a way similar to mastering chess (Bialystok et al., 2012).

Although there is abundant evidence supporting this bilingual advantage, quite a few recent studies have also questioned its existence (e.g., Morton and Harper, 2007; Hilchey and Klein, 2011; Paap and Sawi, 2014; Paap et al., 2015). Paap and Sawi (2014), for example, compared highly proficient bilinguals and monolinguals on tasks that require conflict resolution, attention, and shifting. Across the three tested cognitive control processes, there was no evidence for a bilingual advantage. These and several similar findings have led some researchers to claim that the bilingual advantage does not exist, and the inconsistent results have caused a lively debate about the correctness of the bilingual advantage hypothesis (see Barac et al., 2014, for a review). To make things even more complex, in a meta-analysis on the issue, de Bruin et al. (2015) showed that the bilingual advantage is a reliable effect across studies, but also, taking into account non-published reports, that a publication bias exists against null-findings. This publication bias was confirmed by a recent meta-analysis of Lehtonen et al. (2018). Before correcting for the bias, the authors observed a very small bilingual advantage for conflict resolution, shifting, and working memory. However, no evidence for a bilingual advantage remained after controlling for the publication bias.

Woumans and Duyck (2015) suggested that research on the bilingual advantage should move away from the rather unfruitful debate of whether or not the advantage exists. According to these authors, bilingualism may lead to an advantage in cognitive control, but only for some bilingual profiles. Future work should therefore aim to define the precise characteristics of bilingualism that may benefit cognitive control. Bilingual experience can vary in several ways. For example, bilinguals have varying levels of L2 proficiency, they can differ in their language switching frequency, or in the age at which they acquired their L2. One of these many characteristics that can vary across bilinguals might be the key to enhanced cognitive control. Several other researchers made similar claims (e.g., Prior and Gollan, 2011; Green and Abutalebi, 2013; Woumans et al., 2015; Verreyt et al., 2016). According to the adaptive control hypothesis (Green and Abutalebi, 2013), for instance, the interactional context in which bilinguals use their languages is important. Specifically, those bilinguals who use their languages within the same context (i.e., dual-language context) require a high level of cognitive control to keep their languages separated. This is less true for bilinguals who use their languages in different contexts (i.e., single-language context) or for bilinguals who mix their languages within a sentence or conversation (i.e., dense code-switching context). Using multiple languages in a dual-language context might thus require and hence, train cognitive control processes more than using these languages in single-language or dense code-switching contexts. This hypothesis has been corroborated by recent work showing that bilinguals in dual-language contexts outperform bilinguals in single-language contexts in cognitive flexibility (Hartanto and Yang, 2016). Another factor that has been recently suggested as crucial for the development of a bilingual advantage, is the frequency at which bilinguals switch between their languages (e.g., Prior and Gollan, 2011; Woumans et al., 2015; Verreyt et al., 2016). That is, those bilinguals who switch more frequently between their languages may show more cognitive control advantages than those who switch less often. Language switching requires adaptations in language control (reactivating and inhibiting languages), which each time involves the recruitment of cognitive control. The frequent recruitment of cognitive control for language switching might then train this mechanism. In their study, Verreyt et al. (2016) compared two groups of highly proficient bilinguals (non-frequent and frequent language-switchers) and a group of low proficient bilinguals on conflict resolution. They found a bilingual advantage for the frequent language-switchers over the other groups. These results were further supported by a study of Woumans et al. (2015), who observed a positive correlation between language-switching frequency and conflict resolution. It should be noted, however, that other studies did not obtain evidence for moderating effects of characteristics like language-switching frequency on the bilingual advantage (Yim and Bialystok, 2012; Paap et al., 2017). The bilingual advantage debate therefore continues, and further research and data points are mandatory to understand which specific aspects of bilingualism might lead to enhanced cognitive control. Prior work nevertheless suggests that the bilingual advantage is more likely to emerge in those bilinguals who use their languages in a dual-language context and who switch regularly between their languages. In other words, if a bilingual advantage exists, those bilinguals who require higher levels of language control are more likely to develop it.

What is arguably the most demanding type of bilingualism in terms of language control is simultaneous interpreting. Interpreters have to comprehend incoming speech in a source language and reformulate (translate) this message in the target language, while simultaneously producing a previously translated message. Thus, they have to speak in one language while processing, manipulating and storing considerable amounts of incoming information in the other language. It is estimated that interpreters are speaking in one language while simultaneously comprehending in the other language about 70% of the time (Chernov, 1994). This contrasts with everyday bilingual language practice in which bilinguals typically use only one language at a time. Furthermore and importantly, the languages may not be mixed. The quality of simultaneous interpreting depends in part on the output in the target language. A non-target language intrusion might thus have more negative consequences for interpreters than for other bilinguals, making efficient language control extremely important. This high level of language control requires several cognitive processes (conflict resolution, attention, updating, and short-term memory) to be used in parallel under heavy time pressure (Christoffels et al., 2006; Köpke and Nespoulous, 2006). As language control is assumed to develop cognitive control, expertise in simultaneous interpreting could thus cause interpreters to become experts in several cognitive control processes (Yudes et al., 2011).

Relatively little is known today about the effects of proficiency in simultaneous interpreting on language control, or, more generally, cognitive control. First, there are some inconsistent results regarding the bilingual advantage for interpreters with respect to the cognitive control processes that are often linked to bilingualism (see Table 1 for an overview). In a study of Christoffels et al. (2006), professional interpreters and L2 teachers performed similarly on a basic cognitive control task measuring attention. Yudes et al. (2011) found that professional interpreters outperformed both bilinguals and monolinguals on cognitive flexibility, but not on conflict resolution. An advantage in conflict resolution for interpreters was, however, found by Woumans et al. (2015). In their study, monolinguals, unbalanced bilinguals, balanced bilinguals, and student interpreters were compared. They observed that all bilingual groups outperformed monolinguals on speed of conflict resolution. Furthermore, student interpreters were more accurate than unbalanced, but not than balanced bilinguals. The latter results provide support for the bilingual advantage hypothesis by showing that being highly proficient in multiple languages yields cognitive control advantages, at least in conflict resolution. However, the results of Woumans and colleagues also suggest that experience in simultaneous interpreting may not lead to accumulated advantages in conflict resolution over and above the advantages proper to bilingualism. Morales et al. (2015) found that professional interpreters were better in updating than highly proficient bilinguals, but again they found no difference in terms of conflict resolution. These results provide further support for the finding that professional interpreters have no accumulated advantage in conflict resolution. Experience in simultaneous interpreting might, however, lead to better updating abilities relative to other bilinguals. Therefore, Henrard and Van Daele (2017) compared professional interpreters, translators and monolinguals on a wide range of cognitive control processes (conflict resolution, updating, working memory, speed of information processing, and flexibility). Professional interpreters and translators are both highly proficient bilinguals who have to translate a message from a source language into a target language. However, interpreting is an online process under important time pressure, as interpreters have to comprehend, translate and produce simultaneously. This is not the case for translators, who can process the information in the source language before reformulating the message in the target language. Furthermore, interpreters require a lot of cognitive resources in parallel, as they have to translate while processing a lot of incoming information. Translators, on the other hand, sequentially process the incoming information, translate the message, and produce the output, which requires less cognitive resources. Interpreters therefore might deliberately ignore less relevant information to cope with the time pressure and have to update their memory more than translators. The results of Henrard and Van Daele showed that both bilingual groups outperformed the monolinguals on all cognitive control measures. Moreover, interpreters performed better than translators on all cognitive control aspects, except shifting. Together, these results suggest that experience in interpreting stimulates cognitive control abilities. Research conducted thus far is, however, inconclusive about which cognitive control processes might be specifically enhanced and whether or not there are accumulated advantages for interpreters over other bilingual populations.

**Table 1:** Overview of the studies on cognitive control abilities of interpreters.

Paper	Tested cognitive control process	Participants	Main results
Christoffels et al. (2006)	(i) Attention (ii) STM	Professional interpreters L2 teachers Bilingual students	(i) Students outperformed interpreters on attention (ii) Interpreter advantage at the level of STM
Yudes et al. (2011)	(i) Cognitive flexibility (ii) Conflict resolution (ii) STM	Professional interpreters Bilinguals Monolinguals	(i) Interpreter advantage at the level of cognitive flexibility and STM; bilinguals = monolinguals (ii) No group differences at the level of conflict resolution
Woumans et al. (2015)	Conflict resolution	Student interpreters Monolinguals Unbalanced bilinguals Balanced bilinguals	(i) All bilingual groups better conflict resolution in terms of reaction times (ii) Interpreters more accurate than unbalanced, but not than balanced bilinguals
Morales et al. (2015)	(i) Updating	Professional interpreters	(i) Interpreter advantage in updating
	(ii) Conflict resolution	Highly proficient bilinguals	(ii) No group differences in conflict resolution
Henrard and Van Daele (2017)	(i) Conflict resolution	Professional interpreters	(i) Bilingual groups outperformed monolinguals on all tested cognitive control measures (ii) Interpreters outperformed translators on all cognitive control measures, except on cognitive flexibility
	(ii) Speed of information processing	Translators	
	(iii) Cognitive flexibility	Monolinguals	
	(iv) Updating		
Rosiers et al. (2017)	Conflict resolution	Student interpreters	No evidence for a bilingual advantage in conflict resolution
		Student translators	
		Students multilingual communication	
Liu et al. (2004)	STM	Professional interpreters	No group differences in STM
		Beginner student interpreters	
		Advanced student interpreters	
Köpke and Nespoulous (2006)	(i) Conflict resolution	Professional interpreters	(i) No group differences in STM and conflict resolution (ii) Novice interpreters advantage in working memory
	(ii) STM	Student interpreters	
	(iii) Working memory	Students	
		Bilinguals	


There are also some studies that examined the effects of interpreting on other cognitive control aspects, such as short-term memory (STM; Padilla et al., 2005; Christoffels et al., 2006; Signorelli et al., 2011; Timarova et al., 2014; Rosiers et al., 2017). STM refers to the cognitive system to memorize information (e.g., digits) for a brief period of time (Kolb and Wishaw, 2009). The importance of STM for simultaneous interpreting makes intuitive sense. As noted earlier, interpreters have to temporarily memorize information in the source language while translating it in the target language. Christoffels et al. (2006) found that interpreters performed better on STM tasks than both highly proficient L2 teachers and younger unbalanced bilingual students. Other studies, however, failed to find support for better STM in professional interpreters (e.g., Liu et al., 2004; Köpke and Nespoulous, 2006). Liu et al. (2004), for example, found that student interpreters had similar STM as professional interpreters, despite the fact that the professionals excelled the students in interpreting skills. This finding suggests that accumulating expertise in interpreting does not further train STM. The performance of the student and professional interpreters was not compared to a monolingual control group. This leaves open the question whether or not simultaneous interpreting training develops STM. In another study, Köpke and Nespoulous (2006) assessed the STM of professional interpreters, second-year interpreting students, and two control groups (students and bilinguals). While their data indicated that student interpreters outperformed the control groups, this was not true for professional interpreters who had at least 4 years of professional experience. While these two studies suggest that professional interpreters might not have better developed STM than monolinguals or other bilinguals, they might also be explained by other factors. The authors argued that an effect of expertise in simultaneous interpreting may have been obscured by a confounding effect of age, for example. Nevertheless, observing better or similar performance in STM for student interpreters than for professional interpreters is rather remarkable if simultaneous interpreting relies heavily on STM that further develops with accumulating experience. In their correlational study, Timarova et al. (2014) also only observed a weak association between STM and expertise in simultaneous interpreting. These findings suggest that STM may not be strongly taxed upon during interpreting.

## The Present Study

This study aims to investigate whether special, advanced expertise in L2 benefits cognitive control. We therefore assessed the performance of professional interpreters and L2 teachers on multiple aspects of cognitive control that have been linked to bilingualism. The selection of the different processes was based on the scientific findings about cognitive control in bilinguals and interpreters described above. One major difference between the present study and prior work on interpreters, though, is that we brought all the different aspects of cognitive control together in one study, in multiple groups of advanced L2 users. Indeed, of the relatively few studies examining the cognitive abilities of interpreters, the majority focused on only one or two cognitive control processes (for an exception, see Henrard and Van Daele, 2017).

In Experiment 1, we used three extensively used cognitive control tasks to compare conflict resolution and STM between professional interpreters and monolinguals. Friedman and Miyake (2004) proposed that different conflict resolution tasks may reveal different results because they rely on different conflict resolution types. Two types may be important for bilingualism. Resistance to interference is a type of conflict resolution that allows an individual to focus on the task at hand and to avoid distraction from irrelevant information. Interpreters must resist from being distracted not only by the co-activation of the non-target language, just like typical bilinguals, but also by distractions such as the incoming information in the source language, which competes for attentional resources with the message they are formulating. Furthermore, given that both of their languages have to be active in parallel, interpreters may experience more dual-language competition than typical bilinguals. The second conflict resolution type is prepotent response inhibition. Automatic responses can be caused by developed routines (automatized behavior), or by a triggering response. Bilinguals need prepotent response inhibition to avoid using false cognates, for example. False cognates are word-forms that exist in both languages, but that have a different meaning in each language (e.g., the English-Dutch *room*, which is cream in Dutch). A typical example of this type of conflict resolution in the context of interpreting is the postponement of reformulating (translation) until sufficient information is available to allow for planning (e.g., to avoid interpreting errors caused by syntactic ambiguous sentences). If simultaneous interpreting trains conflict resolution, we anticipate interpreters to outperform monolinguals on both conflict resolution types. We also compared STM of interpreters and monolinguals. As already noted, the ability to temporarily memorize a considerable amount of information is very important for simultaneous interpreting. Furthermore, bilingualism may also lead to better STM (e.g., Grundy and Timmer, 2016). We therefore predict interpreters to have a better STM than monolinguals.

In Experiment 2, we further tested the bilingual cognitive control advantage by introducing a third group of participants, namely L2 teachers. L2 teachers are, like professional interpreters, highly proficient bilinguals, but, as the monolinguals, they have no experience in simultaneous interpreting. They can therefore be considered as typical, highly proficient bilinguals. Assessing different cognitive control processes within the same groups of interpreters and comparing their performance to that of L2 teachers and monolinguals will allow us to determine which aspects of cognitive control are specifically developed by bilingualism and by simultaneous interpreting, more particularly.

### Experiment 1

In Experiment 1, we compared professional interpreters who had at least 4 years of professional experience to monolinguals, using three well-established tasks previously found to be sensitive to the bilingual cognitive control advantage. First, we used the flanker task (Eriksen and Eriksen, 1974) to measure interference suppression. Costa et al. (2008), for instance, found an advantage in interference suppression, reflected in smaller flanker congruency effects for bilinguals than for monolinguals. In their study, the attention network task (ANT) was used, which is a flanker task embedded in a cue reaction time task. It explores three attentional networks, namely cognitive control, alerting, and orienting. With respect to the cognitive control component, which is relevant here, congruent trials were comprised of a target and a flanking arrow pointing in the same direction, whereas a target arrow pointing in one direction and flanking arrows pointing in the other direction were presented on incongruent trials. The difference between congruent and incongruent trials (flanker congruency effect) was taken as a marker of interference suppression.

Second, we assessed prepotent response inhibition with the Simon task (Simon and Wolf, 1963). In this task, participants respond on the color (green or red) of the stimulus, using either their left or right hand, while ignoring its location (left or right). The Simon task includes congruent and incongruent conditions, as this task is based on stimulus-response compatibility. The difference between congruent and incongruent trials (Simon effect) is a marker of prepotent response inhibition. As for the flanker congruency effect, some prior work has reported smaller Simon effects for bilinguals than for monolinguals (e.g., Bialystok et al., 2004) and for interpreters than for monolinguals (Woumans et al., 2015).

Finally, to examine whether simultaneous interpreting improves STM, we used the digit span test. In this task, sequences of digits are presented for immediate serial recall. The length of the sequences gradually increases, making memorization of the sequences more difficult. Some prior work found that bilinguals have a better STM than monolinguals (Bialystok et al., 2008; Morales et al., 2013). Furthermore, there is evidence that interpreters have better STM than various other populations (e.g., Christoffels et al., 2006).

#### Materials and Methods

##### Participants

We recruited 52 participants, divided into two groups (27 monolinguals and 25 interpreters). All participants reported having no language, hearing, uncorrected visual, or neurological problems. Informed consent was obtained under a protocol approved by the ethical committee at Ghent University (Belgium). Objective language proficiency tests could not be used because interpreters had different languages as native language (L1) and L2. Given that self-evaluation correlates strongly with objective measures (Marian et al., 2007; Luk and Bialystok, 2013), participants self-rated their language proficiency. Further, we administered the short untimed 12 item-version of the Advanced Progressive Matrices (Bors and Stokes, 1998) as a measure of intelligence. This version has a strong correlation with the complete 48 item-version (Raven et al., 1998).

Detailed demographic information is reported in Table 2. The 27 monolinguals spoke French as L1 and acquired anecdotal knowledge of an L2 (Dutch or English) through formal education. That is, they indicated having low proficiency in L2 and rarely used this language. The 25 interpreters had at least 4 years of professional experience in simultaneous interpreting. They spoke a variety of languages as L1 (23 Dutch, 1 Portuguese, and 1 French) and L2 (1 Dutch, 2 German, 5 French, 1 Danish, 9 English, 1 Portuguese, and 6 Spanish), but they were all highly proficient in Dutch and used this language for their profession.

**Table 2:** Demographic data of the different participant groups of Experiment 1.

	Monolinguals	Interpreters	Test	*BF*_10_
N	27	25		
Male/female ratio	5/22	9/16	χ^2^(1) = 2.20	0.78
Age (years)	48.37 (8.54)	49.76 (7.99)	*t < 1*	*0.32*
Raven (raw score)	7.37 (2.76)	8.36 (2.12)	*t*(50) = -1.44	*0.65*
L1 proficiency (20-point scale)	19.52 (1.40)	19.96 (0.20)	*t*(27.15) = -1.62	*0.76*
L1 Age of acquisition (years)	0.00 (0.00)	0.08 (0.40)	*t*(24.00) = 1.00	*0.43*
L1 frequency of use (%)	95.56 (6.94)	66.07 (13.68)	*t*(34.97) = 9.68^∗∗∗^	*>100*
L2 proficiency (20-point scale)	5.07 (6.01)	17.44 (1.98)	*t*(31.99) = -10.12^∗∗∗^	*>100*
L2 Age of acquisition (years)	14.11 (7.42)	11.16 (6.16)	*t*(50) = 1.55	*0.75*
L2 frequency of use (%)	4.44 (6.94)	33.93 (13.68)	*t*(34.97) = -9.68^∗∗∗^	*>100*


*SD are shown between parentheses. BF_*10*_, Bayes factor in favor of the alternative hypothesis. ^∗^*p* < 0.05; ^∗∗^*p* < 0.01; *^∗∗∗^p* < 0.001.*

*T*-tests comparing the demographic information between the monolinguals and interpreters are reported in Table 2. We relied on Bayes factors (*BF*_10_) for interpreting our results. Null-hypothesis (H0) significance tests and their accompanied *p*-values have several shortcomings and more reliable alternative approaches, such as *BF*_10_, have been suggested (Gallistel, 2009; Dienes, 2011; Nuzzo, 2014). *BF*_10_ compares the fit of the data under H0 (there is no effect) compared to the alternative hypothesis (there is an effect; H1). *BF*_10_ thus provides a quantification of the degree to which the data support either hypothesis. Values greater than 1 indicate increasing evidence for H1 over H0, values smaller than 1 the reverse. We relied on the guidelines proposed by Jeffreys (1961) for interpreting *BF*_10_ (see Table 3). The monolinguals were matched to the interpreters in terms of age (substantial evidence), male/female ratio (anecdotal evidence), intelligence (anecdotal evidence), and L1 proficiency (anecdotal evidence). As expected, there was decisive evidence that interpreters had a higher proficiency in their L2 than monolinguals and that interpreters used their L2 more frequently than monolinguals.

**Table 3:** Interpretation of Bayes Factors (BF_10_) as evidence for null hypothesis (H0) and alternative hypotheses (H1).

*BF*_10_	Support for hypothesis
<0.01	Decisive evidence for H0
0.03–0.01	Very strong evidence for H0
0.10–0.03	Strong evidence for H0
0.33–0.10	Substantial evidence for H0
0.33–1	Anecdotal evidence for H0
1	No evidence
1–3	Anecdotal evidence for H1
3–10	Substantial evidence for H1
10–30	Strong evidence for H1
30–100	Very strong evidence for H1
>100	Decisive evidence for H1


##### Stimuli and procedure

Participants were tested individually in a quiet room. They were asked to carry out the intelligence test, two computerized cognitive control tasks (flanker task and Simon task), and the digit span task in a counterbalanced order. Task instructions were given in French for monolinguals and in Dutch for interpreters, because monolinguals were recruited in French-speaking Belgium, and interpreters in Dutch-speaking Belgium.

###### Flanker task

The stimuli were white arrows on a black screen that were flanked by four white distractor arrows. The distractor arrows could either point in the same (congruent) or the opposite (incongruent) direction as the target arrow (e.g., congruent trial <<<<< and incongruent trial <<><<).

The task was programmed using Tscope (Stevens et al., 2006). Participants were asked to indicate the direction of the central arrow by pressing the left (a) or right (p) button on an azerty keyboard. Each trial began with a centered 500 ms fixation cross, followed by the stimulus for 1500 ms or until a response was made. There was 500 ms inter trial interval. The task began with 10 practice trials with feedback, followed by two blocks of 100 trials. Each block contained an equal amount of randomly presented congruent and incongruent trials.

###### Simon task

Participants saw colored dots on the left or right side of the screen. They were asked to indicate as quickly and accurately as possible whether the dot was green or red by pressing the left (right) or right (left) key on the keyboard, respectively. Response mapping was counterbalanced across participants. Position and color elicited either the same (congruent trials) or different responses (incongruent trials).

The task was presented via Tscope (Stevens et al., 2006). Each trial began with a 500 ms fixation cross, followed by a 500 ms blank screen. Next, a red or green dot appeared on the left or right side of the screen for 1500 ms or until a response was made, followed by a 500 ms inter trial interval. The task started with 10 practice trials with feedback, followed by two blocks of 100 trials. Each block contained an equal amount of randomly presented congruent and incongruent trials.

###### Digit span task

Series of two to nine numbers (one to nine) were presented in ascending order, with two trials per sequence length. Each number in a sequence was orally presented at a rate of 1000 ms. At the end of a sequence, participants were asked to immediately recall the sequence. A sequence was scored as correct if the sequence was repeated in its correct serial order. Sequences were presented in French for monolinguals and in Dutch for interpreters. The task ended when two trials at a particular sequence length were incorrectly reproduced. The number of correctly recalled sequences was calculated (maximum score: 16).

#### Results

Incorrect responses and outliers were excluded for all analyses on reaction times (RTs). Outlier RTs were trimmed individually by calculating a mean RT for each condition and excluding responses that had an RT of 2.5 *SD* from this mean. Unless stated otherwise, data were analyzed by fitting generalized mixed-effects models with maximum likelihood estimation on individual trials, using the glmer function from the lme4 package in R (Bates et al., 2015). Models on RT data assumed an Inverse Gaussian distribution, and a linear relationship between the predictors and RT (Lo and Andrews, 2015). We initially applied the simplest model, which included the fixed effects, their interactions and the random effect of participants. We included by-participant random slopes if conducted maximum likelihood model comparisons showed that the data justified their inclusion. Planned comparisons were performed using the multcomp package (Hothorn et al., 2008). To calculate *BF*_10_ for main and interaction effects, we used the Bayesian Information Criteria technique (Wagenmakers, 2007). For planned comparisons, we used Bayesian *t-*tests with a default Cauchy prior width of *r* = 0.707 for effect size on H1 (Rouder et al., 2009).

##### Flanker task

**FIGURE 1:**
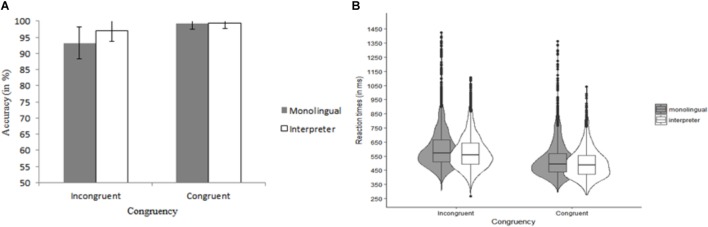
Data of the flanker task as a function of Group (monolingual and interpreter) and Congruency (congruent and incongruent). **(A)** Summarizes the accuracy data. The reaction time data are shown in **(B)**. Error bars denote SE.

The data of one monolingual were excluded because he had an ACC of less than 50% (chance-level) on congruent trials. The ACC data are shown in Figure 1A. For ACC, the model included Group (monolingual, interpreter), Congruency (congruent, incongruent) and their interaction as fixed effects, Participant as random effect and by-Participant random slopes of Congruency. We observed decisive evidence for a main effect of Congruency, χ^2^(1) = 26.58, *p* < 0.001, *BF*_10_ > 100 (flanker congruency effect). There was anecdotal evidence against an effect of Group, χ^2^ < 1, *BF*_10_ = 0.39, and against an interaction of Congruency and Group, χ^2^(1) = 1.52, *p* = 0.22, *BF*_10_ = 0.78.

Of the RT data, 2.45% (248 trials) were outliers. The number of outlier RT trials was similar for the interpreters (*n* = 125) and the monolinguals (*n* = 118), *t* < 1. The trimmed RT data are summarized in Figure 1B. The same model as for ACC data was used for analyzing RTs. We observed decisive evidence for an effect of Congruency, χ^2^(1) = 42.54, *p* < 0.001, *BF*_10_ > 100 (flanker congruency effect). There was very strong evidence against an effect of Group, χ^2^ < 1, *BF*_10_ = 0.02, and against an interaction of Congruency and Group, χ^2^ < 1, *BF*_10_ = 0.01.

##### Simon task

**FIGURE 2:**
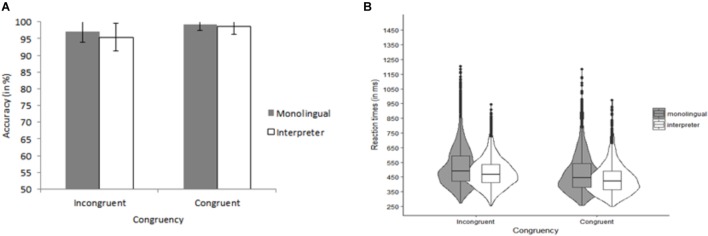
Data of the Simon task as a function of Group (monolingual and interpreter) and Congruency (congruent and incongruent). **(A)** Summarizes the accuracy data. The reaction time data are shown in **(B)**. Error bars denote SE.

Figure 2A summarized the ACC data. For ACC, the model included Group (monolingual and interpreter), Congruency (congruent and incongruent) and their interaction as fixed effects, and Participant as random effect. We observed decisive evidence for a main effect of Congruency, χ^2^(1) = 83.86, *p* < 0.001, *BF*_10_ > 100 (Simon effect). There was strong evidence against an effect of Group, χ^2^(1) = 3.61, *p* = 0.06, *BF*_10_ = 0.06, and against an interaction of Congruency and Group, χ^2^ < 1, *BF*_10_ = 0.01.

Of the RT data, 2.65% (269 trials) were outliers. The number of excluded trials was similar for interpreters (*n* = 136) and monolinguals (*n* = 133), *t* < 1. Figure 2B shows the trimmed RT data. The model on RT contained Group (monolingual and interpreter), Congruency (congruent and incongruent) and their interaction as fixed effects, Participant as random effect and by-Participant random slopes of Congruency. There was decisive evidence for an effect of Congruency, χ^2^(1) = 37.10, *p* < 0.001, *BF*_10_ > 100 (Simon effect), and strong evidence against an effect of Group, χ^2^(1) = 3.49, *p* = 0.06, *BF*_10_ = 0.05. There was very strong evidence against an interaction of Congruency and Group, χ^2^ < 1, *BF*_10_ = 0.01.

##### Digit span task

**FIGURE 3:**
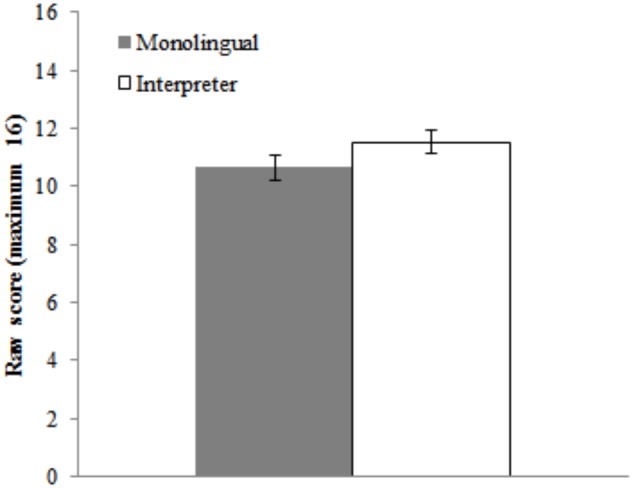
Mean raw scores for the digit span task as a function of Group (monolingual and interpreter). Error bars denote SE.

Digit span performance is summarized in Figure 3. An independent samples *t*-test revealed anecdotal evidence against a group difference in digit span performance, *t*(50) = -1.49, *p* = 0.14, *BF*_10_ = 0.69.

#### Summary of Results

If simultaneous interpreting modulates the bilingual advantage, we would predict better cognitive control for the interpreters. Our data did, however, not reveal evidence for a difference between interpreters and monolinguals on any of the tested cognitive control measures. That is, there were no differences on the flanker congruency effect, indicating similar interference suppression. We also could not observe a group difference on the Simon effect, indicating similar prepotent response inhibition. Finally, the two groups had comparable performance on the digit span task, indicating similar STM. Note, however, that the lack of evidence in favor of group differences was accompanied by decisive evidence for a flanker congruency effect and for a Simon effect. This indicates that the tasks were valid, and sufficiently sensitive, to measure the underlying cognitive control processes.

One might argue that we could not obtain evidence for an interpreter advantage over monolinguals on conflict resolution and STM because interpreters use a different language control mechanism than other, more typical bilinguals (Yudes et al., 2011). It is beyond doubt that language control is more important for interpreters than for other bilinguals, but the specific cognitive control processes involved to achieve language control may differ. There is evidence that both languages are active in parallel in the mind of interpreters and that interpreters therefore experience interference of the non-target language while speaking, just like other bilinguals (Rodriguez-Fornells et al., 2005; Kaushanskaya and Marian, 2007). Interpreters may, however, differ from more typical bilinguals in how they manage cross-language activation. Bilinguals are assumed to select the appropriate language and avoid non-target language interference by inhibiting the latter language (Green, 1998; Dijkstra and Van Heuven, 2002). Interpreters, however, have to maintain both languages active in parallel, one for comprehension and one for speaking. There are indeed some indications that interpreters do not use inhibition to control their languages (Ibáñez et al., 2010), but it is still unknown how interpreters then manage their languages. Nevertheless, if bilinguals and interpreters control their two languages differently, this can lead to differences in (some) cognitive control abilities. That is, simultaneous interpreting may train different aspects of cognitive control than more typical bilingualism. The scope of Experiment 2 was therefore to investigate how the potential cognitive control advantages for interpreters differ from cognitive control advantages associated with more typical bilingual language use. We therefore again examined whether professional interpreters have cognitive control advantages over monolinguals, but we assessed more cognitive control processes that are important for simultaneous interpreting (conflict resolution, attention, STM, and updating). Furthermore, we additionally compared the performance of both groups on each cognitive control measure to that of L2 teachers.

### Experiment 2

In experiment 2, we further investigated the cognitive implications of simultaneous interpreting. We compared professional interpreters with a well-matched group of L2 teachers, based on the following methodological considerations. First, both professional interpreters and L2 teachers are rather rare populations that have very high levels of L2 proficiency. Both populations use their languages for their profession, which makes them frequent language-switchers in a dual-language context. Finally, they also share a similar educational background, as they both have a degree in L2 and share an interest in language. One important difference between interpreters and L2 teachers, though, is the amount of interpreting experience they have, and therefore the amount of language control training that can be expected. It is reasonable to assume that interpreters require higher levels of language control than L2 teachers. There may also be qualitative differences between the cognitive control processes involved in language control, which can lead to differences between interpreters and L2 teachers on these cognitive control abilities. Interpreters have to resolve conflict, store considerable amounts of information in STM, and update their memory, without confusing their languages. Assessing these different cognitive control processes within the same groups of interpreters and comparing their performance with that of L2 teachers and monolinguals will allow us to determine which cognitive control aspects are specifically developed by bilingualism and by experience in simultaneous interpreting, more particularly.

The advanced flanker task (Emmorey et al., 2008) was used to measure two types of conflict resolution, as it is a combination of the flanker task and the go/no-go task, measuring resistance to interference and prepotent response inhibition, respectively. We anticipate a bilingual advantage in both conflict resolution types. If interpreting involves resistance to interference or prepotent response inhibition, we also predict interpreters to outperform L2 teachers because of accumulated practice. Conversely, if interpreters do not use inhibition for language control, in contrast to L2 teachers, we predict L2 teachers to outperform both monolinguals and interpreters. Additionally, the advanced flanker task allowed us examining another cognitive control aspect, namely attention. It almost goes without saying that high levels of attention are important during interpreting, as it enables an individual to speak, listen, and manipulate information simultaneously. We therefore anticipate interpreters to outperform both L2 teachers and monolinguals in attentional abilities.

The third cognitive control process assessed was STM, using Hebb learning. Hebb learning is an immediate serial recall task in which sequences of items (e.g., phonemes) are presented. We chose this task because phoneme recall is not dependent upon prior language knowledge. This is important because functional STM may not be the same in bilinguals’ L1 and L2 (Service et al., 2002). In Experiment 1, the monolinguals and interpreters carried out the digit span test in different languages, which may have obscured the detection of possible group differences. Given the importance of STM for simultaneous interpreting, we anticipate interpreters to outperform L2 teachers and monolinguals. We also predict L2 teachers to outperform monolinguals, in line with prior work suggesting bilingual advantages on STM (Bialystok et al., 2008; Morales et al., 2013; Grundy and Timmer, 2016). Furthermore, it has been shown that Hebb learning can be considered as an analog of novel word-form learning (e.g., Szmalec et al., 2009; Smalle et al., 2017). When a particular sequence of phonemes is repeated, performance for the repeating Hebb sequence improves relative to non-repeating filler sequences (Hebb, 1961). This finding (Hebb repetition effect) reflects the gradual transfer of newly acquired serial-order information from STM to long-term memory, which underlies novel word learning. Given the indications that bilinguals outperform monolinguals in learning novel words (Kaushanskaya and Marian, 2009; Nair et al., 2017) and that better STM has been associated with superior word learning abilities in bilinguals (Papagno and Vallar, 1995; Kaushanskaya, 2012), we also investigated whether interpreters outperform other groups on the Hebb repetition effect.

The fourth and final aspect of cognitive control tested here was updating, using the *n*-back task (Collette et al., 2001; Oberauer, 2005; Szmalec et al., 2011). A typical feature of STM is that its capacity is limited (Cowan, 2005). Thus, when confronted with a large stream of incoming information, individuals must temporarily store subsets of information and successively update STM as more information becomes available. This is exactly what needs to be done during simultaneous interpreting: a continuous stream of incoming information in the source language needs to be temporarily held in STM while it is being reformulated in the target language, and then “forgotten” in order to store and reformulate new information in the source language. We therefore predict interpreters to have better updating abilities than both L2 teachers and monolinguals. Given that prior research has shown that bilinguals outperform monolinguals in updating (e.g., Bialystok et al., 2006), we also anticipate L2 teachers to have better updating abilities than monolinguals.

To summarize, using three tasks we examined the possibilities of bilingual advantages in interpreters at the level of interference suppression, prepotent response inhibition, attention, STM, and updating. We also tested whether interpreters have advantages at the level of the Hebb repetition effect, an analog of novel word learning. We not only investigated whether interpreting leads to improved cognitive control over monolinguals, but also how the cognitive implications of simultaneous interpreting may differ from more typical bilingual language use. Based on the research explained above and assuming the existence of a bilingual advantage, we predict L2 teachers and interpreters to outperform monolinguals on all cognitive control measures. If the bilingual advantage is specifically related to extensive language control, interpreters are anticipated also to outperform L2 teachers.

#### Materials and Methods

##### Participants

A total of 59 participants were recruited and divided into three groups: 19 professional interpreters, 20 L2 teachers, and 20 monolinguals. All participants reported having no language, hearing, uncorrected visual, or neurological problems. Informed consent was obtained under a protocol approved by the ethical committee at the Université catholique de Louvain, Belgium. As for Experiment 1, objective language proficiency tests could not be used given that interpreters had different languages as L1 and L2. Participants filled in the Language Experience and Proficiency Questionnaire (LEAP-Q) to obtain self-rated language proficiency (Marian et al., 2007). Further, as in Experiment 1, we administered the short untimed 12 item-version of the Advanced Progressive Matrices (Bors and Stokes, 1998) as a measure of intelligence.

Detailed demographic information is reported in Table 4. All groups were highly proficient in French. The 20 monolinguals had French as L1 and acquired anecdotal knowledge of an L2 (Dutch or English) through formal education. That is, they indicated that they had low L2 proficiency and rarely used this language (see Table 3). The 20 L2 teachers were highly proficient bilinguals with no experience in simultaneous interpreting. They spoke French (*n* = 18) or Dutch (*n* = 3) as L1 and had at least 4 years of experience in teaching L2 courses (English or French). The 19 interpreters had at least 4 year of professional experience in simultaneous interpreting. They spoke a variety of languages as L1 (8 French, 4 Dutch, 4 English, 1 German, and 2 Spanish) and L2 (6 French, 2 Dutch, 6 English, 1 Italian, 1 Spanish, 1 German, 1 Polish, and 1 Russian).

**Table 4:** Demographic data of the different participant groups in Experiment 2.

	Monolinguals	L2 teachers	Interpreters	Test	*BF*_10_
N	20	20	19		
Male/female ratio	4/16	4/16	6/13	χ^2^ < 1	*0.14*
Age (years)	44.40 (8.30)	44.15 (8.43)	48.58 (9.76)	*F*(2, 56) = 1.53	*0.28*
Education (years)	16.45 (2.70)	17.45 (3.78)	18.42 (1.84)	*F*(2,56) = 2.25	*0.51*
Raven (raw score)	7.80 (2.02)	7.20 (2.61)	8.05 (1.96)	*F* < 1	*0.15*
L1 proficiency (10-point scale)	8.80 (0.58)	9.33 (0.60)	9.61 (0.59)	*F*(2, 56) = 9.64^∗∗∗^	*>100*
L1 age of acquisition (years)	0.75 (0.64)	0.90 (2.27)	1.74 (3.63)	*F* < 1	*0.17*
L1 frequency of use (%)	95.20 (6.43)	59.15 (15.70)	44.89 (14.13)	*F*(2,56) = 81.62^∗∗∗^	*>100*
L2 proficiency (10-point scale)	2.25 (2.16)	8.33 (1.43)	8.68 (0.62)	*F*(2,56) = 107.71^∗∗∗^	*>100*
L2 age of acquisition (years)	12.35 (2.50)	8.10 (5.17)	11.05 (6.21)	*F*(2,56) = 4.02^∗^	*2.02*
L2 frequency of use (%)	3.80 (4.92)	29.15 (12.38)	32.95 (13.29)	*F*(2,56) = 42.40^∗∗∗^	*>100*


*SD are shown between parentheses. ^∗^*p* < 0.05; ^∗∗^*p* < 0.01; *^∗∗∗^p* < 0.001.*

The three groups were matched on age (substantial evidence), male/female ratio (substantial evidence), years of education (anecdotal evidence), and intelligence (substantial evidence). Planned comparisons showed anecdotal evidence that interpreters and L2 teachers were matched on L1 and L2 proficiency, *t*(37) = 1.15, *p* = 0.15, *BF*_10_ = 0.73 for L1 and *t*(26.25) = -1.00, *p* = 0.34, *BF*_10_ = 0.46 for L2. Furthermore, both L2 teachers and interpreters reported higher L1 proficiency than the monolingual group [monolinguals vs. teachers: *t*(38) = -2.86, *p* < 0.01, *BF*_10_ = 6.68 (substantial evidence); monolinguals vs. interpreters: *t*(37) = -4.36, *p* < 0.001, *BF*_10_ > 100 (decisive evidence)]. The same was true for L2 proficiency [monolinguals vs. teachers: *t*(38) = -10.94, *p* < 0.001, *BF*_10_ > 100 (decisive evidence); monolinguals vs. interpreters: *t*(22.29) = -12.76, *p* < 0.001, *BF*_10_ > 100 (decisive evidence)].

##### Stimuli and procedure

Participants were tested individually in a quiet room. They were asked to carry out the intelligence test and three computerized tasks (advanced flanker task, *n*-back task, Hebb repetition paradigm) in a counterbalanced order.

###### Advanced flanker task

**FIGURE 4:**
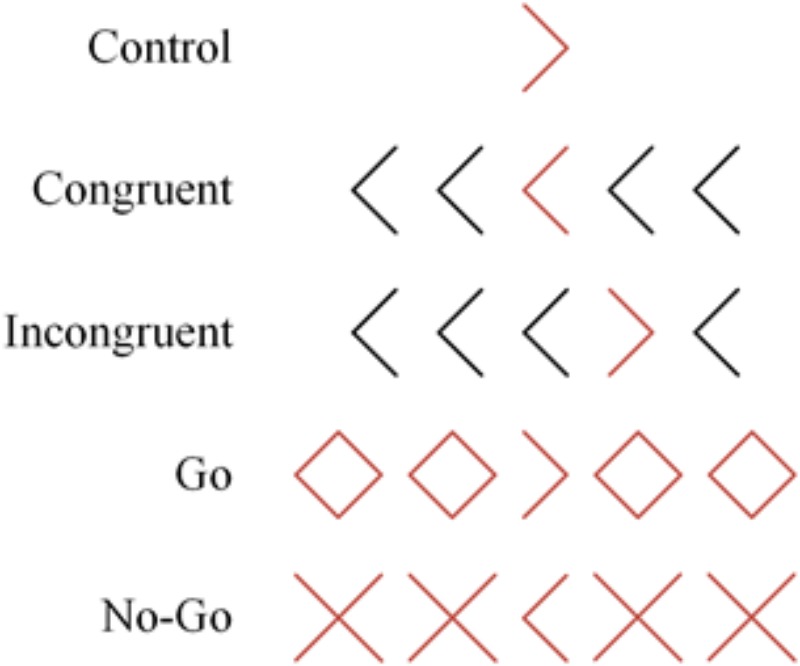
Examples of the different trial types of the advanced flanker task, adapted from Emmorey et al. (2008).

The stimuli were red arrows that could be flanked by four distractors (Figure 4). There were three block types. In control blocks, participants saw single red arrows pointing to the left or right. These blocks provide a measure of attention. In flanker blocks, there was an equal number of congruent (flanking black arrows pointing in the same direction as the red target arrow) and incongruent trials (flanking black arrows pointing in the opposite direction as the red target arrow). On incongruent trials, participants had to inhibit interference of the flanking arrows. The difference in performance between congruent and incongruent trials (i.e., flanker congruency effect) reflects a measure of interference suppression. The red arrow could be either presented in the middle or one place to the left or right of the middle position. This was done to prevent participants from focusing solely on the middle stimulus. Finally, in go/no-go blocks, there were an equal proportion of go and no-go trials. On go trials, a central red arrow was flanked by four red diamonds, two on each side. Participants had to indicate the direction of the red arrow as fast as possible. On no-go trials, the arrow was flanked by four red Xs and participants were required to withhold their responses. In this go/no-go block, participants were required to inhibit their responses on no-go trials while responding as rapidly as possible on go trials. The difference in performance between go and no-go trials (i.e., go/no-go congruency effect) provides a measure of prepotent response inhibition.

The task was programmed using Tscope (Stevens et al., 2006). Participants were asked to indicate the direction of the red arrow by pressing the left (d) or right (k) button on a keyboard. Each trial began with a centered 250 ms fixation cross, followed by the stimulus for 2000 ms or until a response was made. There was an inter-trial interval of 500 ms. Each block type was presented twice. Control blocks were always presented as the first and last blocks, with flanker and go/no-go blocks alternating between them in a counterbalanced order. Each block began with 12 practice trials with feedback, followed by 48 trials. Trial types were randomized within each block.

###### Hebb repetition paradigm

The materials and procedure were based on the study of Szmalec et al. (2009). Sequences of nine syllables with a consonant-vowel structure were presented visually to the participants for immediate serial recall. Two sets (A and B) of nine syllables that were matched on bigram frequency (in French) were generated using WordGen (see Table 5; Duyck et al., 2004). For half of the participants, set A was used for filler sequences and set B for the Hebb sequence. For the other half it was the reverse. Overall task performance was taken as a measure of STM. The Hebb repetition effect (i.e., the different performance for Hebb and filler trials) provides a measure of long-term memory sequence learning that has been shown to underlie novel word-form acquisition.

**Table 5:** Syllables and their French bigram frequency used in the Hebb repetition paradigm.

	1	2	3	4	5	6	7	8	9
Set A	VE	DA	FI	GU	JO	ZI	WA	XA	RO
	1416	892	1153	889	253	99	36	104	3642
Set B	CO	CU	MI	BI	PE	JI	MU	PO	XU
	3821	957	1885	1202	1537	8	477	1833	44


The task was developed in E-prime 2.0 (Psychology Software Tools, Pittsburgh, PA, United States). Syllables were presented sequentially for 1000 ms. There was an inter-syllable interval of 500 ms. After the presentation of the sequence, a recall screen was presented on which all syllables were randomly positioned in a circle around a central question mark. Participants were instructed to click with the computer mouse on the syllables in the same order in which they were presented. Participants could click the question mark to indicate an omission, at the position in the sequence of the forgotten syllable. This way, correct responses after an omission are still in the right serial position. When participants clicked nine times (on syllables or the question mark), they were asked to press the space bar to start the following trial. The task started with two practice trials. Participants always saw two consecutive filler sequences, followed by the Hebb sequence. The experiment ended when the participant correctly reproduced two successive Hebb trials, with a maximum of 20 repetitions.

###### *N*-back task

A *2*-back version was used. Participants saw a long sequence of items and were asked to indicate for each individually presented item whether it was the same as the one that was presented *2* positions before (an example of a match is t–d–m–d; a mismatch is t–h–m–d). Participants were thus required to remember the 2 most recently presented items in their correct serial order. This implied that they had to update the memorized sequence of the 2 most recent items after each trial. On lure trials, a word did not match the word that was presented 2 items before, but one of its neighboring items (an example of an *n* + 1 lure **d**–h–m–d; an *n*-1 lure is t–h–**d**–d). Lure trials typically lead to slower responses and reduced accuracy (McElree, 2001; Gray et al., 2003; Oberauer, 2005; Jonides and Nee, 2006; Kane et al., 2007; Szmalec et al., 2011). This is because continuously updating items in STM hinders distinguishing between relevant and irrelevant items. Although the entire task is an updating task, lure interference effects (i.e., the difference between mismatch and lure trials) were taken as a measure of updating abilities, because recollection demands are most strongly involved in lure trials. On these trials, participants must make a clear distinction between the current trial (requiring a negative response) and the previous trial (which would lead to a positive response). If updating is not efficient, this should lead to larger lure interference effects.

The procedure and materials were held as close as possible to Szmalec et al. (2011). Participants were asked to indicate as quickly as possible whether or not the presented consonant on the screen matched the item that was presented 2 consonants earlier, by pressing the right (k) or left (d) button on a keyboard, respectively.

The task was developed in E-Prime 2.0. Each trial started with the presentation of a 500 ms consonant, followed by a 2500 ms fixation cross. The task consisted of 20 practice trials that did not contain lure trials, followed by four randomly presented blocks of 45 + 2 (two stimuli that did not require a response at the beginning of each list of consonants) trials. Each block contained 15 match trials, 24 mismatch trials, 3 *n*-1 lure trials, and 3 *n* + 1 lure trials that were presented in a pseudo-random order.

#### Results

The same data-analyses procedures as in Experiment 1 were used.

##### Advanced flanker task

**FIGURE 5:**
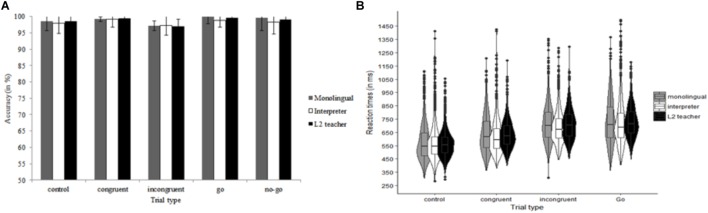
Data for the advanced flanker task. **(A)** Shows accuracy data as a function of Group (monolingual, interpreter, and L2 teacher) and Trial type (control, congruent, incongruent, and go, no-go). **(B)** Summarizes the reaction time data as a function of Group (monolingual, interpreter, and L2 teacher) and Trial type (control, congruent, incongruent, and go). Error bars denote SE.

The data of one L2 teacher was excluded because he had an average ACC of only 50% (chance-level) for congruent trials. The ACC data are shown in Figure 5A. The final model on ACC for the control block contained Group (interpreter, L2 teacher, monolingual) as fixed effect and Participant as random effect. The model on ACC for the Go/no-go block contained Group (interpreter, L2 teacher, and monolingual) and Trial type (go and no-go) and their interaction as fixed effects, Participant as random effect and by-participant random slopes of Trial type. The final model on ACC for the analyses for the flanker block contained Group (interpreter, L2 teacher, and monolingual) and Congruency (congruent and incongruent) as fixed effects, Participant as random effects and by-Participant random slopes of Congruency. For the control block, we observed decisive evidence against a main effect of Group, χ^2^ < 1, *BF*_10_ < 0.01. For the Go/no-go block, there was very strong evidence against a main effect of Group, χ^2^(2) = 8.08, *p* = 0.02, *BF*_10_ = 0.01, and substantial evidence against an effect of Trial type, χ^2^(1) = 5.59, *p* = 0.02, *BF*_10_ = 0.22. There was decisive evidence against an interaction of Group and Trial type, χ^2^ < 1, *BF*_10_ < 0.01. For the flanker block, we observed decisive evidence against an effect of Group, χ^2^ < 1, *BF*_10_ < 0.01. There was decisive evidence in favor of an effect of Congruency, χ^2^(1) = 37.55, *p* < 0.001 *BF*_10_ < 0.01 (i.e., flanker congruency effect). There was decisive evidence against an interaction of Group and Congruency, χ^2^ < 1, *BF*_10_ < 0.01.

2.26% of the RT data (310 trials) were outliers. A univariate ANOVA indicated that there were no differences between the number of trials excluded for the interpreters (*n* = 105), the monolinguals (*n* = 108), and the L2 teachers (*n* = 97), *F* < 1. The trimmed RT data are summarized in Figure 5B. The final model on RT for the control and Go/no-go block contained Group (interpreter, L2 teacher, and monolingual) as fixed effect and Participant as random effect. The final model on RT for the analyses for the flanker block contained Group (interpreter, L2 teacher, and monolingual) and Congruency (congruent and incongruent) as fixed effects, Participant as random effects and by-Participant random slopes of Congruency. For both the control and go block, we observed decisive evidence against a main effect of Group, χ^2^ < 1, *BF*_10_ < 0.01. For the flanker block, there was decisive evidence for a main effect of Trial type, χ^2^(1) = 61.47, *p* < 0.001, *BF*_10_ > 100 (i.e., flanker congruency effect). There was decisive evidence against a main effect of Group, as well as against an interaction of Congruency and Group, χ^2^ < 1, *BF*_10_ < 0.01 for both effects.

##### Hebb repetition paradigm

**FIGURE 6:**
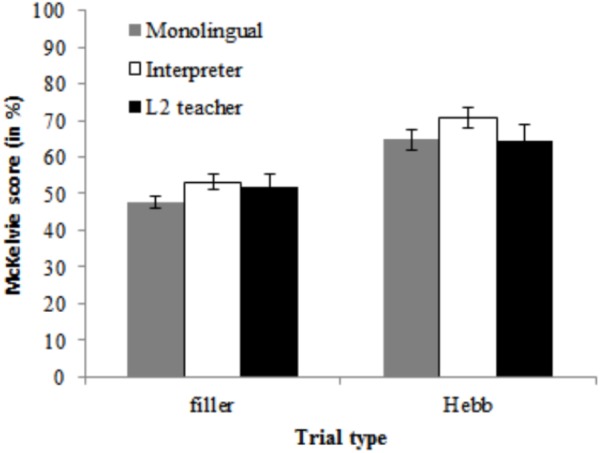
McKelvie scores for the Hebb repetition paradigm as a function of Group (monolingual, interpreter, and L2 teacher) and Trial type (filler and Hebb). Error bars denote SE.

Hebb recall performance was calculated with the McKelvie scoring method (McKelvie, 1987). This method takes into account both the position and serial order of recalled items. First, we counted the number of items that were in the correct position from left to right up to the first error. Second, the same step was repeated from right to left up to the first error. Third, the number of items in any correct sequence of two or more items between the first error from the left and the first error from the right was counted. Finally, any other items that occurred in the correct serial position were counted. The maximal possible score for each sequence was 9. The mean McKelvie score for each Group and each Trial type are presented in Figure 6. Analyses were performed at the mean level, because not all participants had the same number of trials due to the stopping criterion. The model on the McKelvie scores included Group (monolingual, L2 teacher, and professional interpreter), Trial type (filler and Hebb) and their interaction as fixed effects, and Participant as random effect. We observed decisive evidence for an effect of Trial type (i.e., Hebb repetition effect), χ^2^(1) = 82.82, *p* < 0.001, *BF*_10_ > 100, but strong evidence against a main effect of Group, χ^2^(1) = 2.24, *p* = 0.33, *BF*_10_ = 0.03. There was also strong evidence against an interaction of Trial type and Group, χ^2^(2) = 3.28, *p* = 0.19, *BF*_10_ = 0.04. On average, the monolingual group needed 14.50 repetitions (*SD* = 5.72) to reach the stopping criterion, the L2 teachers 12.60 repetitions (*SD* = 6.89) and the interpreters 14.63 repetitions (*SD* = 5.89). A univariate ANOVA on the number of repetitions showed that there was substantial evidence against a group difference, *F* < 1, *BF*_10_ = 0.13.

##### *N*-back task

**FIGURE 7:**
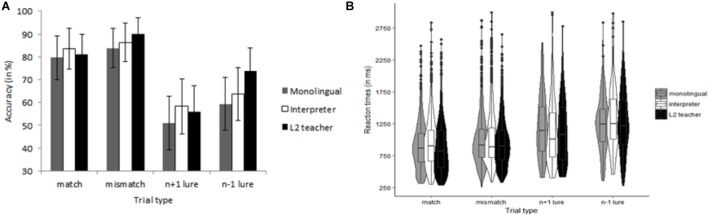
Data for the *n*-back task as a function of Group (monolingual, interpreter, and L2 teacher) and Trial type (match, mismatch, *n* + 1 lure, and *n*–1 lure). **(A)** Summarizes the accuracy data. The reaction time data are shown in **(B)**. Error bars denote SE.

The data of two monolinguals, one L2 teacher, and two professional interpreters were excluded because they had an ACC below 50% (i.e., chance-level) on match trials. The final sample contained 18 monolinguals, 19 L2 teachers, and 17 interpreters. The ACC data are presented in Figure 7A. For ACC, the model included Group (interpreter, L2 teacher, and monolingual), Trial type (mismatch, match, *n* + 1 lure, and *n*-1 lure) and their interaction as fixed effects, Participant and Trial order as random effects and by-Participant random slopes of Trial type. Trial order was included to control for learning effects, as we presented trials in a counterbalanced order (Baayen et al., 2008). We observed decisive evidence for an effect of Trial type, χ^2^(3) = 76.36, *p* < 0.001, *BF*_10_ > 100. There was decisive evidence against an effect of Group, χ^2^(2) = 1.69, *p* = 0.43, *BF*_10_ < 0.01, and against an interaction of Trial type and Group, χ^2^(6) = 4.08, *p* = 0.67, *BF*_10_ < 0.01. Planned comparisons on Trial type revealed decisive evidence for an *n* + 1 lure effect (mismatch vs. *n* + 1 lures), *t* = -19.46, *p* < 0.001, *BF*_10_ > 100, and for an *n*-1 lure effect (mismatch vs. *n*-1 lures), *t* = -13.43, *p* < 0.001, *BF*_10_ > 100.

The RT data are summarized in Figure 7B. Here, 2.42% of the RTs (192 trials) were outliers. There were no differences between the number of trials excluded for the interpreters (*n* = 52), the monolinguals (*n* = 66), and the L2 teachers (*n* = 74), *F*(51) = 1.77, *p* = 0.18. The same model as for ACC was used for analyses on RTs. We observed decisive evidence for an effect of Trial type, χ^2^(3) = 122.93, *p* < 0.001, *BF*_10_ > 100. There was decisive evidence against an effect of Group, χ^2^ < 1, *BF*_10_ < 0.01, and against an interaction of Trial type and Group, χ^2^(6) = 1.65, *p* = 0.95, *BF*_10_ < 0.01. Planned comparisons on Trial type revealed decisive evidence for a *n* + 1 lure effect, *t* = 7.65, *p* < 0.001, *BF*_10_ > 100, and a *n*-1 lure effect, *t* = 14.68, *p* < 0.001, *BF*_10_ > 100.

#### Summary of Results

The aim of Experiment 2 was to investigate whether the high levels of language control of professional interpreters amplify possible cognitive control advantages often associated with bilingualism. Therefore, we compared three participant groups (professional interpreters, L2 teachers, and monolinguals) on a wide range of cognitive control measures, including interference suppression, response inhibition, attention, STM, and updating. Overall, we did not find support for a bilingual or interpreter advantage. First, our results on the advanced flanker task revealed evidence for similar flanker congruency effects for the three groups. The results on this task also showed that there were no differences between the three groups in the terms of the go/no-go congruency effect or on the control block. Together, these results suggest that all groups had similar performance in terms of interference suppression, prepotent response inhibition, and attention, respectively. Second, the results on the Hebb repetition paradigm also provided strong evidence against group differences. There was no evidence for an overall better performance for L2 teachers or interpreters relative to monolinguals. The interpreters also performed similarly to the L2 teachers. This indicates that all groups had similar STM. Furthermore, the comparable Hebb repetition effect for the three groups suggests that there were no differences in terms of long-term memory sequence learning that underlies novel word-form learning. Finally, there were no differences between the three groups on lure interference in the *n*-back task, indicating similar updating abilities. Thus, we found no evidence for an interpreter advantage on any tested cognitive control aspect. Indeed, our data showed that the interpreters and the L2 teachers performed very similarly on conflict resolution (interference suppression and prepotent response inhibition), attention, STM, and updating. Furthermore, both bilingual groups did not differ from the monolinguals in terms of their cognitive control performance, indicating that there was no measurable bilingual advantage.

It is worth mentioning that the lack of evidence for a bilingual advantage in Experiment 2 was accompanied in each task by decisive evidence in favor of the expected markers of cognitive control. As such, our participants had clear flanker congruency effects in the advanced flanker task, lure effects in the *n*-back paradigm, and clear Hebb repetition effects. This shows that the tasks used in the current study were valid and sensitive to the underlying cognitive control processes that they were meant to measure.

### Cross-Experiment Comparison

**FIGURE 8:**
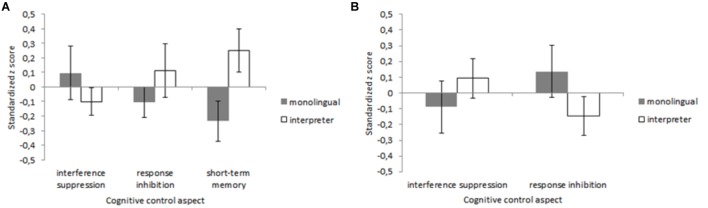
Standardized *z* scores for the accuracy data **(A)** and the reaction times **(B)** of the two experiments as a function of Group (monolingual and interpreter) and Cognitive control aspect (interference suppression, prepotent response inhibition, and short-term memory). Error bars denote SE.

We performed additional analyses to further explore the reliability of our null-findings. Although we obtained similar results in two independent experiments, which strengthens the reliability of our results, there may still be smaller bilingual or interpreter advantages that we were not able to detect. If such small group differences exist, we might detect them by combining the data of Experiments 1 and 2. To this end, we calculated standardized *z* scores for the accuracy data of interpreters and monolinguals for the measures of interference suppression (flanker congruency effect in both experiments), prepotent response inhibition (Simon effect for Experiment 1, go/no go congruency effect for Experiment 2), and STM (digit span task performance for Experiment 1, overall performance on the Hebb task for Experiment 2) for each Experiment. The *z* scores for the ACC data are shown in Figure 8A. We also calculated the *z* scores for the reaction time data of interpreters and monolinguals for the measures of interference suppression (flanker congruency effect in both experiments), and prepotent response inhibition (Simon effect for Experiment 2, go RTs for Experiment 2) for each Experiment. The *z* scores for the RT data are summarized in Figure 8B.

For both the ACC and RT data on interference suppression, independent samples *t*-tests comparing 45 interpreters and 46 monolinguals revealed no evidence in favor of any group differences, *t* < 1, *BF*_10_ = 0.33, and *t* < 1, *BF*_10_ = 0.31, respectively. The same was true for prepotent response inhibition. That is, independent samples *t*-tests comparing 44 interpreters and 47 monolinguals on both the composite score for ACC and RT revealed no evidence in favor of any group differences, *t*(89) = -1.04, *p* = 0.30, *BF*_10_ = 0.35, and *t* < 1, *BF*_10_ = 0.22, respectively. In contrast, comparing the STM data of 44 interpreters and 47 monolinguals, we observed anecdotal evidence for better STM for interpreters than for monolinguals, *t*(89) = 2.40, *p* = 0.02, *BF*_10_ = 2.65. These results suggest that, although only to a small degree, experience in simultaneous interpreting may to some extent be associated with better STM performance.

## Discussion

The main purpose of this study was to investigate whether the high levels of language control of interpreters amplify possible cognitive control advantages often associated with bilingualism. We therefore conducted two experiments in which we compared interpreters to other populations (monolinguals and L2 teachers) on a wide range of cognitive control measures, including conflict resolution, attention, STM, and updating. Based on the adaptive control hypothesis (Green and Abutalebi, 2013), we predicted that the two bilingual groups would outperform the monolingual group on all cognitive control measures. Furthermore, we anticipated that the interpreters would outperform the L2 teachers because the higher language control demands associated with simultaneous interpreting could amplify the bilingual advantage.

In Experiment 1, we used the flanker, Simon, and digit span tasks to compare professional interpreters and monolinguals on interference suppression, prepotent response inhibition, and STM, respectively. We did not find evidence for any cognitive control advantage for interpreters over monolinguals. In Experiment 2, we compared the performance of professional interpreters, L2 teachers and monolinguals on interference suppression, prepotent response inhibition, attention, STM, and updating. We therefore used an advanced flanker task, an *n*-back task and a Hebb repetition paradigm. Again, we did not observe support for a bilingual or interpreter advantage on any of the measures. The combined results of Experiment 1 and 2 indicate that the interpreters performed like the monolinguals and the L2 teachers on all the tested cognitive control processes. This suggests that there is no bilingual advantage in cognitive control, at least not for L2 teachers and interpreters. To further examine this result, we conducted an additional set of analyses. By merging the data of both experiments by analyzing the standardized composite scores in a cross-experiment comparison, we found further support that experience in simultaneous interpreting does not lead to an advantage in conflict resolution, neither at the level of interference suppression, nor at the level of prepotent response inhibition. The cross-experiment analyses did, however, reveal a small but measurable advantage for interpreters over monolinguals in terms of STM. Given that we had no group of L2 teachers in Experiment 1, we were not able to test whether the STM advantage was related to bilingualism or specifically to simultaneous interpreting. In sum, the combined results of Experiment 1 and 2 that the interpreters performed like the monolinguals and the L2 teachers suggests that there is no bilingual or interpreter advantage at the level of conflict resolution, attention, and updating. The results provide, on the other hand, anecdotal evidence for a (small) bilingual advantage in STM.

The fact that we have not found empirical support for the existence of an advantage for our bilingual groups on most of the tested cognitive control processes (conflict resolution, attention, and updating) is noteworthy. We examined highly proficient interpreters who all had at least 4 years of professional experience and may therefore be assumed to have extensive training in language control. Furthermore, we also recruited highly proficient L2 teachers who were using their languages daily for their professional activities. Our results therefore suggest that neither using languages in a dual-language context, nor having extensive training in language control is a guarantee to develop overall superior cognitive control abilities. The current findings are in line with previous studies that failed to obtain evidence for better performance for bilinguals than for monolinguals on multiple aspects of cognitive control (Paap and Greenberg, 2013; Paap et al., 2015) or for professional interpreters in particular (e.g., Liu et al., 2004; Christoffels et al., 2006; Yudes et al., 2011; Babcock and Vallesi, 2017).

**FIGURE 9:**
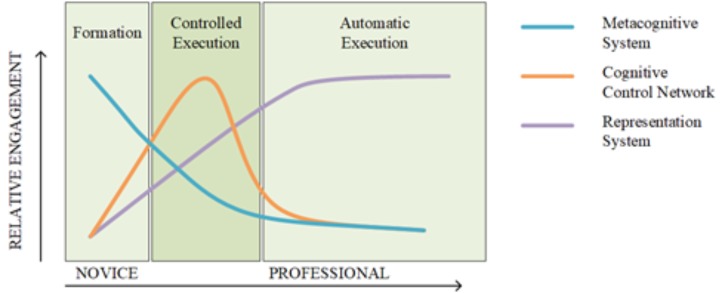
The framework of behavioral learning of Chein and Schneider (2012).

There are several possible explanations for the lack of enhanced conflict resolution, attention, and updating for interpreters relative to other groups. First, as already noted, interpreters may not use their language control mechanisms as other, more typical bilinguals. Interpreters arguably experience more cross-language interference between their languages and a greater requirement to produce the correct output in the target language. Given these extreme language control demands, interpreters might develop qualitative different methods to manage their languages and to be able to comprehend and produce information in different languages simultaneously. Consequently, interpreters might not develop better cognitive control, because they are not using the same language control mechanisms as other bilinguals. Nevertheless, we also could not observe an advantage for L2 teachers over monolinguals, suggesting that even for more typical bilinguals there might not be a bilingual advantage. This brings us to the second possibility, which is that there is no bilingual advantage at the level of conflict resolution, attention, updating, and novel word learning. In a recent study, Van de Putte et al. (2018) also did not find support for the hypothesis that interpreting experience enhances cognitive control. In their study, interpreters and translators performed similarly on tasks measuring conflict resolution and shifting, both before and after a 9-month training in their profession. However, only after the language training, the authors observed increased activation for the interpreters relative to the translators in the right angular gyrus during the shifting task and in the left superior temporal gyrus during the conflict resolution task. As neural measures were outside the scope of the current study, future work should shed light on the relationship between simultaneous interpreting training, the associated neural changes, and their relation to behavioral cognitive control measures. Third, it should be mentioned that the monolinguals tested in the current study also acquired (passive and anecdotal) knowledge of a L2. It cannot be excluded that the interpreters and L2 teachers tested here improved their cognitive control abilities, but that the improvement is not linearly related to L2 proficiency or that they reached a ceiling. That is, the dual-language use and higher demands of language control might not further increase the cognitive control benefits that all participants already had due to the fact that they all knew a L2. A fourth and final possible explanation for the absence of a bilingual or interpreter advantage on the aforementioned aspects of cognitive control is that our bilingual groups were too proficient. Paap (2018) proposed the Controlled Dose hypothesis, which states that the bilingual advantage might only be present during the process of L2 acquisition. This hypothesis is based on a general framework of behavioral learning proposed by Chein and Schneider (2012). The acquisition of novel behavior typically proceeds with shifting from relying on the metacognitive system during the formation stage, to recruiting the cognitive control network during the controlled-execution stage and, finally, to relying on the representation system during the automatic-execution stage (see Figure 9). According to the Controlled Dose hypothesis (Paap, 2018), there might be a similar shift in engagement of cognitive control for bilinguals. The bilingual advantage may therefore only be present during a particular period of L2 acquisition, when bilinguals are still learning how to juggle their languages. Once bilinguals have sufficient training in language control, language management might become an automatic skill that does not require cognitive control processes. Similar to losing better developed muscles when stopping physical fitness training, the benefits in cognitive control of bilinguals might not persist indelibly when this mechanism is no longer recruited for language control. This hypothesis is new and still needs to be investigated. According to the Controlled Dose hypothesis, a benefit in cognitive control might thus be predicted for interpreters and L2 teachers, but these advantages are likely to be transitory. Less experienced interpreters and L2 teachers who are still becoming more proficient in their job may still train their cognitive control with every linguistic choice they make, so that there can be a cognitive control advantage for these populations.

Prior research has indeed found bilingual advantages for interpreters that were still at the start of their professional career. Woumans et al. (2015), for instance, compared the conflict resolution performance of student interpreters, student balanced bilinguals, student unbalanced bilinguals, and student monolinguals. They used the Simon task to measure prepotent response inhibition and the ANT to measure interference suppression. All bilingual groups had a smaller Simon effect than the monolinguals, suggesting better prepotent response inhibition. Furthermore, both the interpreters and balanced bilinguals had a smaller congruency effect on the ANT, indicating superior interference suppression. It is possible that an advantage was found in the study of Woumans et al. (2015), but not in the current study, because of the fact that the student interpreters and student balanced bilinguals in the study of Woumans et al. (2015),were still gaining L2 proficiency, whereas the bilingual groups tested here were not. This idea also fits with the Bilingual Expertise hypothesis (Incera and McLennan, 2016; Damian et al., 2018). It has been found that proficient bilinguals take longer to start moving the mouse in a mouse tracking paradigm but then move more efficiently than monolinguals to the correct response. However, no group differences emerge in terms of RTs. It is thus possible that bilinguals change the way in which they approach cognitive control tasks once they have sufficient training in language control, although this does not imply better performance. Our study design does not permit to draw any firm conclusions about the Controlled Dose hypothesis, but together with the findings of prior research it shows that it deserves further investigation. The bilingual profile of the participants in studies of this type should be controlled carefully in the future, as it would enable us to understand when bilingualism provides an advantage in cognitive control and why.

Regardless of the explanation, the results of the current study indicate that neither high levels of L2 proficiency and use in a dual-language context, nor experience with simultaneous interpreting leads to measurable enhancements in conflict resolution, attention, and updating. We did, on the other hand, find some evidence for improved STM for interpreters when compared to monolinguals. Although our findings should be interpreted with caution given the anecdotal evidence in favor of its existence, this interpreter advantage at the level of STM is in line with previous studies which have shown that simultaneous interpreters have better STM when compared to other populations (Bajo et al., 2000; Padilla et al., 2005; Christoffels et al., 2006; Signorelli et al., 2011; Yudes et al., 2011; Stavrakaki et al., 2012; Babcock and Vallesi, 2017). Christoffels et al. (2006), for instance, examined whether professional interpreters had better STM than bilingual university students and highly proficient L2 teachers. They recruited 13 interpreters, 39 bilingual students, and 15 L2 teachers. Using a word span task that was highly comparable to the digit span task used in Experiment 2, they observed that interpreters outperformed both the students and the L2 teachers, while the students and the L2 teachers performed similarly. The authors also found that interpreters, bilingual students and L2 teachers performed similarly on a basic reaction time task, measuring attention. The authors therefore concluded that working memory is a crucial cognitive control aspect for simultaneous interpreting, whereas attention is not. In the current study, we were not able to find evidence for an interpreter advantage on the digit span task, despite the fact that we used a highly similar design as in the study of Christoffels and colleagues and that we tested groups of comparable size. Nevertheless, the results of the cross-experiment comparison did reveal (small) evidence for an interpreter advantage, in line with the findings of Christoffels et al. (2006). The fact that the advantage in working memory was rather small is further in line with a recent meta-analysis of Grundy and Timmer (2016). In their study, they analyzed the advantage in STM for bilinguals over monolinguals combining data from 88 effect sizes, 27 independent studies, and 2901 participants. Their results revealed a small to medium effect in favor of a bilingual advantage in working memory. So, it appears that bilingualism can give an advantage in working memory, but this advantage is rather small and may therefore be difficult to detect. Across all other cognitive control measures, on the other hand, we found no evidence in favor of an interpreter or bilingual advantage. Together, the current findings therefore further corroborate to the idea that simultaneous interpreting may lead to enhanced STM relative to monolinguals, albeit that this advantage is rather small. An advantage in STM for interpreters over other populations is reasonable given the nature and demands of simultaneous interpreting. Working memory is a crucial component because interpreters have to store content in a source language and reformulate this content in the target language while articulating previous reformulated messages. This high working memory demand appears to alter STM capacity.

The present results should be regarded with a degree of caution, as there are certain limitations worth noting. First, the monolingual group in Experiment 2 had a lower L1 proficiency than the two bilingual groups. This contrasts with prior work, which found that bilinguals have reduced vocabulary knowledge in their L1 relative to monolinguals (Bialystok et al., 2009). The higher L1 proficiency of the bilinguals tested here is likely due to the fact that both professional interpreters and L2 teachers are linguists that received formal education in their L1, which was not true for the monolinguals. This could have influenced the results for our measure of novel word learning. Nevertheless, even with this advantage in L1 proficiency, there were still no differences between the bilinguals and the monolinguals in the Hebb repetition paradigm. Second, in Experiment 2, we compared the performance of interpreters, L2 teachers, and monolinguals on tasks that were selected because they appeal on particular cognitive control processes (conflict resolution, attention, updating, working memory, long-term memory consolidation that underlies word learning). The choice of our tasks raises some questions. First, overall accuracy on the conflict resolution tasks was high. Bialystok (2015) argued that the bilingual advantage is more likely to emerge in more effortful tasks. Although accuracy rates are comparable to past research that did obtain evidence for a bilingual advantage (e.g., Emmorey et al., 2008), it cannot be excluded that the tasks were not sufficiently effortful to detect differences in conflict resolution between our groups. Furthermore, with respect to the *n*-back task, it would also be interesting to examine whether memory updating is better for interpreters or bilinguals in general if words are used instead of consonants. Remembering and updating words is more naturalistic and is more in line with the professional activities of interpreters than consonants. Finally, we decided to use a visual version of the Hebb repetition paradigm. Given the nature of simultaneous interpreting, it would be interesting to examine in future work whether an oral version of the Hebb repetition paradigm, in which the sequences are not presented visually but auditory, elicits better performance for interpreters. During their profession, interpreters hear incoming information which they have to transform and story in their memory. Previous research that reported an interpreter advantage mainly used oral working memory tasks (e.g., Padilla et al., 2005; Christoffels et al., 2006; Signorelli et al., 2011). Nevertheless, the digit span task in Experiment 1 was an oral STM task, where we also failed to observe strong evidence for an interpreter advantage. It should be noted, though, that the testing language of this task was different for monolinguals and interpreters. Although Dutch and French digit names are very similar in terms of worth length, it cannot be ruled out that cross-language variability in digit span performance masked possible group differences. Nevertheless, although simultaneous interpreting is likely to train specifically oral STM, the current study suggests that this advantage is rather small.

To conclude, the results of the current study once more point toward the complexity of the phenomenon of bilingualism and the difficulty to determine its cognitive implications. Prior work has suggested that particular characteristics of bilingualism might be important for the advantage to emerge. The amount of language control needed in daily life has been proposed as being the modulating factor. The results of this study provide further insights in this matter by showing that extensive training in language control does not necessarily always lead to general beneficial effects on cognitive control. Although we found ambiguous evidence that interpreters have better STM than monolinguals, there was no evidence for an advantage at the level of conflict resolution, attention, updating, and novel word learning. Further research is needed to determine whether there might be a certain period during language control training in which cognitive control is (overall) enhanced. Comparing the bilingual advantage between novice and professional interpreters in a longitudinal design could shed more light on the (temporary) importance of cognitive control in the bilingual brain.

## Data Availability Statement

The original datasets and R code of the different tasks for this study can be found at Mendeley data (doi: 10.17632/jcr9yswps8.3).

## Ethics Statement

This study was carried out in accordance with the recommendations of the ethical committee of Ghent University (Belgium) and the ethical committee of the Psychological Sciences Research Institute at the Université catholique de Louvain (Belgium), with written informed consent from all subjects. All subjects gave written informed consent in accordance with the Declaration of Helsinki.

## Author Contributions

LVdL and EVdP contributed to the acquisition of the data. LVdL carried out the analysis and interpretation of the data, the preparation of the figures, and the writing of the manuscript. All co-authors contributed to the conception of the study, the interpretation of the data, and the content and editing of the manuscript.

## Conflict of Interest Statement

The authors declare that the research was conducted in the absence of any commercial or financial relationships that could be construed as a potential conflict of interest.
